# Study rationale and design of OPTIMISE, a randomised controlled trial on the effect of benchmarking on quality of care in type 2 diabetes mellitus

**DOI:** 10.1186/1475-2840-10-82

**Published:** 2011-09-22

**Authors:** Frank Nobels, Noëmi Debacker, Carlos Brotons, Moses Elisaf, Michel P Hermans, Georges Michel, Erik Muls

**Affiliations:** 1 Endocrinologie, OLV Ziekenhuis, Moorselbaan 164, B-9300 Aalst, Belgium; 2 Corilus, Hogenakkerhoer 5 - 9150, Kruibeke, Belgium; 3 Research Unit, EAP Sardenya-IIB Sant Pau, Sardenya, 466. 08025, Barcelona, Spain; 4 Department of Internal Medicine, School of Medicine, University of Ioannina, 451 10 Ioannina, Greece; 5 Endocrinology & Nutrition, Cliniques universitaires St-Luc, DIAB 54.74 Tour Claude Bernard +1 avenue Hippocrate 54, B-1200 Brussels, Belgium; 6 Endocrinology, Centre Hospitalier de Luxembourg, 4 rue Barblé, L-1210 Luxembourg; 7 Department of Endocrinology, UZ Gasthuisberg, Herestraat 49, B-3000 Leuven, Belgium

## Abstract

**Background:**

To investigate the effect of physician- and patient-specific feedback with benchmarking on the quality of care in adults with type 2 diabetes mellitus (T2DM).

**Methods:**

Study centres in six European countries were randomised to either a benchmarking or control group. Physicians in both groups received feedback on modifiable outcome indicators (glycated haemoglobin [HbA_1c_], glycaemia, total cholesterol, high density lipoprotein-cholesterol, low density lipoprotein [LDL]-cholesterol and triglycerides) for each patient at 0, 4, 8 and 12 months, based on the four times yearly control visits recommended by international guidelines. The benchmarking group also received comparative results on three critical quality indicators of vascular risk (HbA_1c_, LDL-cholesterol and systolic blood pressure [SBP]), checked against the results of their colleagues from the same country, and versus pre-set targets. After 12 months of follow up, the percentage of patients achieving the pre-determined targets for the three critical quality indicators will be assessed in the two groups.

**Results:**

Recruitment was completed in December 2008 with 3994 evaluable patients.

**Conclusions:**

This paper discusses the study rationale and design of OPTIMISE, a randomised controlled study, that will help assess whether benchmarking is a useful clinical tool for improving outcomes in T2DM in primary care.

**Trial registration:**

NCT00681850

## Background

Physicians in Europe and throughout the world are diagnosing and treating a growing number of patients with type 2 diabetes mellitus (T2DM). The International Diabetes Federation estimates that approximately 55 million people in Europe had diabetes in 2010 [1]. Over 90% of these are patients with T2DM, and management of its associated complications already requires a significant level of support in terms of financial costs and healthcare utilisation [2]. In 2009 the healthcare expenditure for diabetes was estimated to account for 10% of the total health budget in Europe [1] and this financial burden, together with the accompanying social burden associated with reduced quality of life and productivity caused by the condition, its numerous comorbidities and premature death, is undoubtedly set to rise over the next decade.

Clinical research has shown that long-term complications of diabetes can be delayed or even prevented when management by the healthcare team and the patient's self-management are optimised [3-5]. However, the apparent inability to effectively translate this scientific evidence into real-life clinical practice represents a major barrier to reducing the burden of diabetes complications [6,7].

Western healthcare systems are currently in a transition phase; shifting towards organisational structures that can deliver improved diabetes care specifically, and chronic care in general [8,9]. T2DM is often used as 'the' test case for new models of care. The assumption is that if an intervention works in as complex a disease as T2DM, there is a fair likelihood that it will be effective in other chronic conditions. Because the majority of chronic illness care is performed within the primary care setting, redesign of primary care has been proposed in an attempt to close the gap between current practices and optimal standards [10]. How such a redesign might be accomplished is the subject of ongoing research and socio-economic debate, and in this process the Chronic Care Model is often used as a conceptual framework [11-13]. This model identifies six essential elements that impact upon chronic care: (i) community resources and policies, (ii) healthcare organisation, (iii) self-management support, (iv) delivery system design, (v) decision support, and (vi) clinical information systems [12]. Initial evidence demonstrates that the Chronic Care Model can improve chronic care and, in some cases, reduce healthcare costs [8,9,13]. However, more research is needed to substantiate the relative effectiveness of the components of this model.

One element of the Chronic Care Model that has not been studied extensively, but which nevertheless may offer a realistic opportunity to improve chronic care, is the use of clinical information systems. These include reminder systems that help primary care teams comply with practice guidelines, and feedback to physicians showing how they are performing with regard to quality of care indicators. Practice recommendations (which may be based on national or international guidelines) are generally used as a reference for comparison when providing feedback.

**Figure 1:**
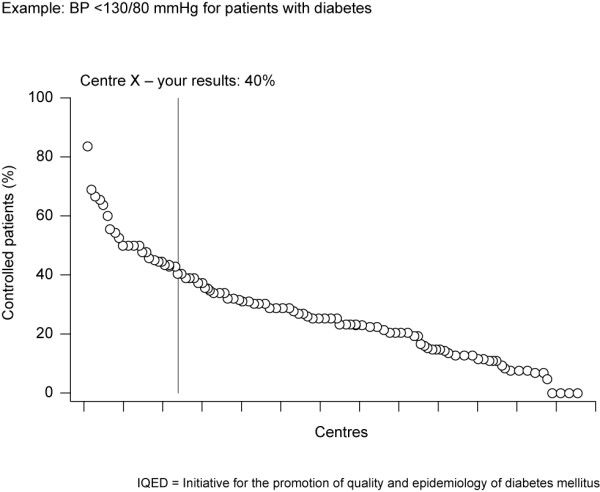
**Example of benchmarking and feedback process**.

Benchmarking, a technique used for quality management in industry since the 1980s, has only recently been introduced into the clinical setting. It consists of measuring the clinical performance of the healthcare delivered by an individual clinician or a clinic over a specified period (termed the audit), comparing it with the performance of a peer group or a group of similar clinics (benchmarking), and transmitting this comparison back to the clinician or clinic (benchmarked feedback) (Figure 1). Benchmarking therefore encompasses an intellectual, emotional and/or competitive stimulus for change. It also offers an opportunity to break through the isolation that many primary care physicians encounter, as they are not routinely able to compare their performance with that of their peers.

It would appear to be intuitively logical that if healthcare professionals were given feedback that their clinical practice, and therefore their patient outcomes, was inconsistent with that of their peers or accepted guidelines, they would modify their attitude and practice. However, there are few published reports of randomised studies investigating this approach in T2DM in primary care, and the effectiveness of feedback and benchmarking has not been definitively proven in a randomised, controlled setting. OPTIMISE (OPtimal Type 2 dIabetes Management Including benchmarking and Standard trEatment) was designed as a randomised controlled trial assessing the effect over a 12-month period of physician- and patient-specific feedback with benchmarking on the quality of primary care in T2DM patients from six European countries. The aim of the current paper is to describe the rationale for, and methodology used in the OPTIMISE study, including details of the benchmarking and feedback process.

## Methods

The original protocol developed in Belgium was subsequently adapted for extension to five other European countries: Greece, Luxembourg, Portugal, Spain and the UK. Patient recruitment began in March 2008 in Belgium, and in September 2008 for the other countries.

### Patients

All patients with T2DM (insulin treated or not), aged ≥18 years were eligible for the OPTIMISE study. Exclusion criteria were patients with type 1 diabetes, gestational diabetes, patients who were hospitalised for diabetes regulation or who were members of the Belgian Diabetes Convention (i.e. hospital-based diabetes centres already participating in a quality assurance programme with benchmarked feedback). The definition of diabetes used was a fasting plasma glucose test of ≥126 mg/dL.

### Study design

This randomised controlled trial was performed in accordance with the Declaration of Helsinki and ICH/Good Clinical Practice and applicable regulatory requirements [14]. The study was sponsored by AstraZeneca. Written, informed consent was obtained for all patients before initiation into the study. The OPTIMISE study is registered at ClinicalTrials.gov (NCT00681850).

The aim of the study was to assess the impact of benchmarking on the quality of T2DM care, by determining, as the primary objective, the percentage of patients in the benchmarking group achieving, after 12 months of follow up, pre-set targets of three critical quality indicators (glycated haemoglobin [HbA_1c_], low-density lipoprotein [LDL]-cholesterol and systolic BP [SBP]) versus the control group. The null hypothesis was that the percentage of patients at target after 12 months of follow up in the benchmarking and the control groups would be equal. Secondary objectives included determining the percentage of patients achieving the pre-set targets of the critical quality indicators and glycaemia versus baseline; determining the percentage improvement of the critical quality indicators and glycaemia versus baseline; and to follow up evolution markers of preventative screening (retinopathy, neuropathy, dietary counselling, microalbuminuria, smoking habits, body mass index [BMI], and physical activity).

**Figure 2:**
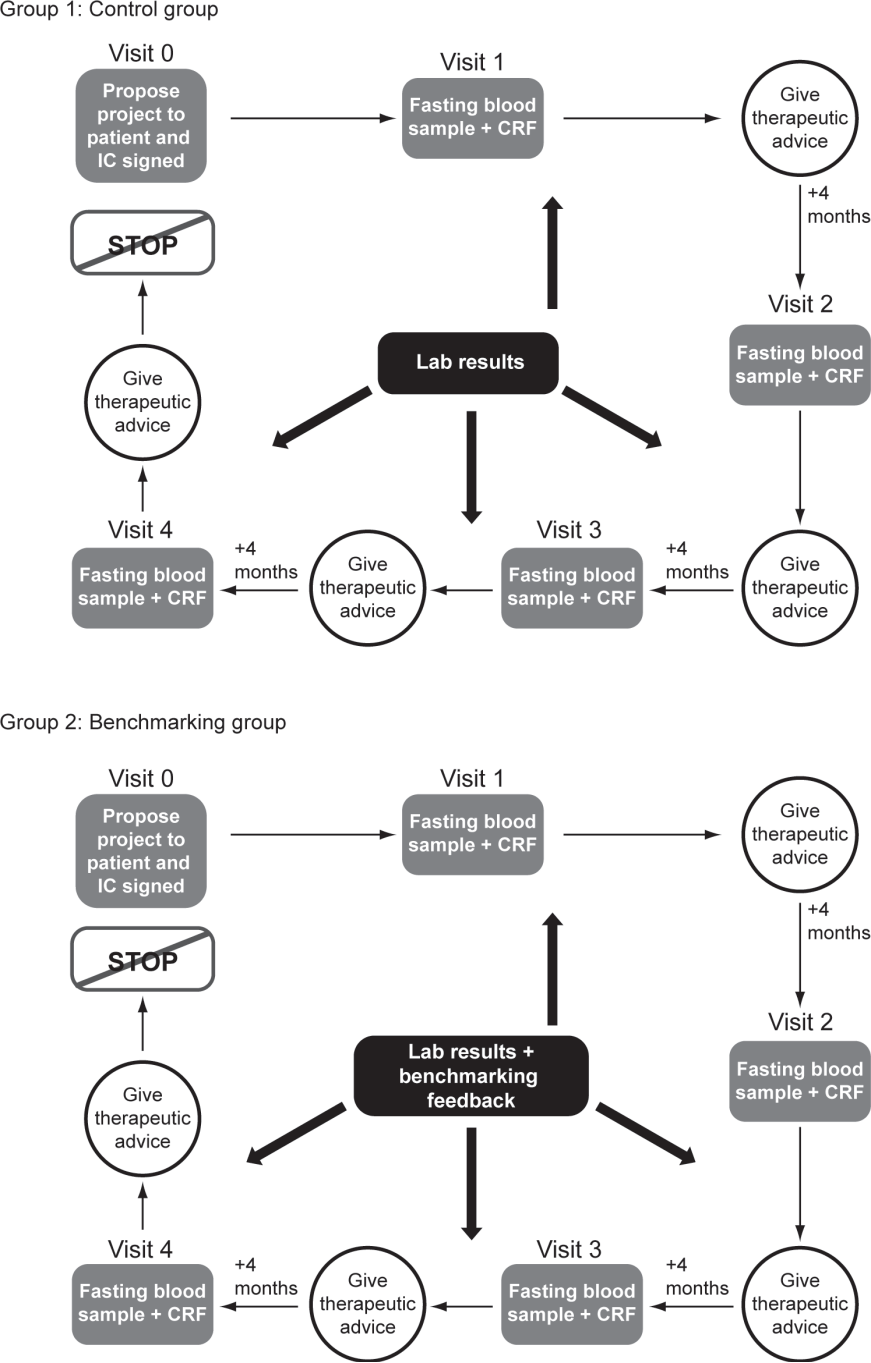
**Study flow chart**. CRF, case report form; IC, informed consent.

Centres or sites were selected on the basis of having sufficient patients with diabetes in their practice and with investigators that were motivated to fulfil the administrative procedures linked to the study. Centres or sites were randomised by a central randomisation procedure to either a benchmarking group or a control group. To ensure that country-specific treatment practices were reflected in the study population, the centres or sites involved in the management of diabetes that were recruited, consisted of general practitioner or hospital-based outpatient clinics. Recruitment for the study began initially in Belgium with a randomisation ratio of 1:1. Each centre was expected to recruit 8-12 patients; however, some centres enrolled 15-20 patients. The adapted sample size analysis showed that a power of 79% was achieved for the parameter blood pressure and a significance level of 0.05 was achieved for the parameters HbA_1c _and total cholesterol. As sufficiently high numbers of physicians were recruited in Belgium, this subsequently allowed a 3:1 randomisation ratio of benchmarked to control practices in the other countries, which was deemed more attractive to the participating physicians. All physicians involved in the study continued with the routine monitoring, treatment and counselling of their patients with T2DM. Over the course of the study, physicians in both groups received the results of modifiable outcome indicators (HbA_1c_, fasting glycaemia, total cholesterol, high-density lipoprotein [HDL]-cholesterol, LDL-cholesterol and triglycerides [TG]; determined in a central laboratory). Physicians in the benchmarking group additionally received information on the level of control of the critical quality indicators of their individual patients, anonymously compared with the results of their colleagues from the same country, and compared with target levels (Figure 2). This benchmarked feedback was provided every 4 months, corresponding to three- to four-times yearly control visits, as recommended by international guidelines [15]. The study recruitment and initiation visit was defined as Visit 1 out of the four visits.

### Patient evaluation and quality indicators

Each patient was followed for 12 months. Overnight fasting levels of HbA_1c_, glycaemia, total cholesterol, HDL- and LDL-cholesterol and TG were determined at each visit. Blood samples were sent to a central laboratory (Bio Analytical Research Corporation [BARC], Ghent, Belgium) for analysis. HbA_1c _was measured using ion-exchange high performance liquid chromatography (HPLC) (Adams A_1c _HA8160, Arkray, Kyoto, Japan). LDL-cholesterol data from evaluable patients was estimated using Friedewald's formula, which uses total cholesterol, HDL-cholesterol and fasting TG when the latter are < 400 mg/dL. Urine was analysed for microalbuminuria at Visits 1 and 4 using an immunoturbidimetric assay (Modular P analyser, Roche Diagnostics, Mannheim, Germany).

Casual (resting) BP, waist circumference, weight and height were measured by the physician at each visit. BP was measured using a manometer with an appropriately sized cuff with the patient in the sitting position for at least 5 minutes prior to the reading. A mean value was determined from three successive readings and recorded. Waist circumference was measured using a measuring tape placed around the patient's abdomen at the level of the iliac crest and at the end of normal expiration. Patients wore light clothing and no shoes during weight measurement - values were rounded up to the nearest kg from 0.5 kg. Patients wore no shoes during height measurement - values were rounded up to the nearest cm from 0.5 cm.

A history of proteinuria at baseline was established from proteinuria levels in the patient's medical records and computed in the electronic Case Report Form (eCRF). Smoking status was also recorded in the eCRF as smoker/ex-smoker/non-smoker, together with the number of cigarettes, cigars or pipes that the patient was smoking, or had smoked per day. In addition, records were kept of whether or not a needs assessment for low-dose aspirin therapy (as an antiplatelet agent) had been performed during the 12 months of the study, as at the time the protocol was written, this was a recommended parameter to be taken into consideration for patients with T2DM [16]. Optional assessment by the treating physician at each visit of the degree of physical activity could be carried out using a step counter. A four-point verbal rating scale (VRS) was used to assess total physical activity with the following criteria; no weekly activity; only limited physical activity during most weeks; intense physical activity (activity that gives rise to shortness of breath, tachycardia and sweating) during at least 20 minutes, once to twice a week; intense physical activity (activity that gives rise to shortness of breath, tachycardia and sweating) during at least 20 minutes, ≥3 times a week.

Critical quality indicators used for benchmarking were HbA_1c_, LDL-cholesterol and SBP. Secondary quality indicators, not used for benchmarking, were blood glucose, TG, total and HDL-cholesterol, diastolic blood pressure (DBP), BMI, waist circumference and smoking status. Whether or not dietary advice had been given (and by whom), and whether screening for retinopathy and foot examination had been performed during the 12 months of the investigation, were also recorded and analysed as secondary quality indicators but were not part of the benchmarking feedback.

### Target setting for benchmarking

The targets set for the critical quality indicators (HbA_1c_, LDL-cholesterol and SBP) used for benchmarking were based on international guidelines [16-18], and, when deemed necessary, adapted by the study steering committee in order to be consensual to the participating countries (Table 1).

**Table 1:** OPTIMISE Study: Targets set for benchmarking

	SBP (mmHg)	LDL-cholesterol (mg/dL)	**HbA**_**1c**_ (%)
All countries	< 130*	< 100^†^	< 7.0
Belgium		< 80^†^	

*Patients with proteinuria < 125 mmHg

^†^Patients with existing coronary heart disease < 70 mg/dL

**Figure 3:**
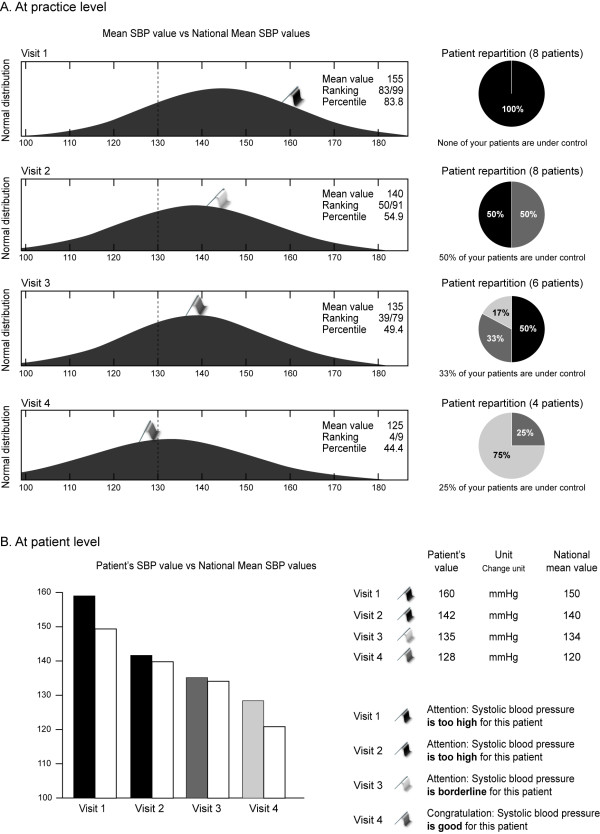
**Example of benchmarking in OPTIMISE**. Reproduced with permission from Whathealth.eu. SBP, systolic blood pressure.

For physicians in the benchmarking group, benchmarked feedback was given to each practice for both the pooled patient population at that practice, and at an individual patient level (Figure 3). A bi-modal response was given (good, too high) for each of the critical quality indicators, signifying whether or not the patient/clinic had achieved targets. In Belgium, the National Steering Committee opted for a tri-modal response: HbA_1c _≤7.0% = excellent, 7.1-7.5% = borderline, > 7.5% = too high; SBP < 130 mmHg = excellent, 130-139 mmHg = borderline, ≥140 mmHg = too high; LDL-cholesterol < 80 mg/dL = excellent, 80-99 mg/dL = good, ≥100 mg/dL = too high (< 70 mg/dL = excellent in secondary prevention).

### Statistical considerations

In this study, all patients treated by an investigator received the same type of follow up (either benchmarking or standard). Since each investigator may have a particular approach for monitoring, counselling and treating their patients with T2DM, it was expected that this might result in an investigator or cluster effect. The extent of this effect is reflected by the Intracluster Correlation Coefficient (ICC) which, according to the literature, varies for BP between 0.05 and 0.10, indicating a clustering influence of 5-10% [19-21]. Of the three critical quality indicators used for benchmarking, SBP was expected to be the least well controlled at baseline. Therefore, for the purposes of study sample size calculations, it was considered most important to be able to demonstrate significance in the improvement in proportion of patients achieving the pre-set targets for SBP.

For example in the Best study [22], blood pressure was controlled in only 12.3% of patients at baseline; however, the proportion of patients with their blood pressure under control increased by 68.3% after 6 months of more intensive follow up. Due to the stricter target (130/80 mmHg in the OPTIMISE study compared with 140/90 mmHg) and the longer duration of this study, an improvement of 88.7% could be expected in the proportion of patients reaching blood pressure targets in both groups, from 12.3% to 23.2% in the control group and the benchmarking group under the null hypothesis (the null hypothesis is that the percentages in both groups are equal). There are relatively few previous studies reporting the level of improvement seen when benchmarking was used. However, review of those available [23-27] suggest that the level of improvement that might be expected ranges from 15-51%. Based on these findings it was estimated that the use of benchmarking in OPTIMISE could further improve the level of blood pressure control by 32%, i.e from 23.2 to 30.6% (the alternative hypothesis is that the percentage in the benchmarking group is larger than in the control group). With 3000 evaluable patients in the benchmarking group (300 clusters of 10 patients) and 1000 patients in the control group (100 clusters of 10 patients), a two-sided Z test (unpooled) would give 91%, 86% and 80% power to detect a 74% difference between the proportion of patients on target for SBP for an ICC of 0.05, 0.07 and 0.10 (between 5 and 10%).

As some centres included up to 20 patients, sample size calculations were adapted. With 3000 evaluable patients in the benchmarking group (200 clusters of 15 patients) and 1000 patients in the control group (67 clusters of 15 patients), there would be 96%, 93% and 88% power to detect a 74% difference between the proportion of patients on target for SBP for an ICC of 0.05, 0.07 and 0.10, respectively, p = 0.05. With 3000 evaluable patients in the benchmarking group (150 clusters of 20 patients) and 1000 patients in the control group (50 clusters of 20 patients), there would be 93%, 88% and 81% power to detect a 74% difference between the proportion of patients on target for SBP for an ICC of 0.05, 0.07 and 0.10, respectively, p = 0.05.

Descriptive statistics (mean, median, number of observations, standard deviation, standard error, 95% confidence intervals - minimum and maximum) of all primary and other variables will be tabulated. Given the expected clustering of patients for each physician, multilevel mixed modelling will be performed using SAS GLINMIX for categorical variables (target attainment after 12 months in the benchmarking group compared with the control group, and target attainment after 12 months compared with baseline levels) and SAS Prox MIX for continuous variables (mean percentage improvement of HbA_1c_, LDL-cholesterol and SBP after 12 months follow up).

## Results

Recruitment commenced on 6 March 2008 and finished 31 December 2008. The database lock date was 31 March 2010. A total of 4026 patients, of which 3994 are evaluable, were enrolled from 370 sites across six countries in Europe. In the All Patients group, 55.1% were male. The benchmarking group comprised 2484 patients; the control group comprised 1510 patients (Table 2). Baseline demographics were similar for both groups. Full results will be reported elsewhere.

**Table 2:** OPTIMISE Study: Number of patients participating in each country

Country	Benchmarking	Control	All patients
Belgium	1146	1044	2190
Greece	570	227	797
Luxembourg	136	62	198
Portugal	134	51	185
Spain	257	55	312
UK	241	71	312
Total	2484	1510	3994

## Discussion

It is important to note that there is some confusion in the literature between the terms feedback and benchmarking. In the OPTIMISE study, benchmarking refers to the process of comparing critical quality indicators with the results of peers. Communicating these results to the physicians will be referred to as benchmarked feedback or feedback with benchmarking, to distinguish it from communicating the levels of the quality indicators alone to the physicians.

There is emerging evidence from non-randomised controlled trials (non-RCTs) that feedback and benchmarking exert a positive effect on process, outcome and overall provision of diabetes care [28-31]. However, follow up over time without specific interventions has also demonstrated improvements in diabetes care [32,33]. Thus, it is difficult in non-RCTs to ascertain whether any detected improvements in care are due to the specific intervention applied.

There is limited evidence from RCTs on the effects of feedback on quality of care in T2DM in particular, and in chronic care in general. Furthermore, evidence from RCTs on the effect of benchmarking is virtually non-existent. A recent systematic literature review [34] identified 10 RCTs evaluating feedback only, but not benchmarking, in T2DM in primary care. The studies were very heterogeneous in aims and designs and a meta-analysis of these trials was not considered feasible. A variety of outcome measures were studied. Feedback in the T2DM studies seemed to improve process markers such as foot examinations, eye examinations and HbA_1c _measurements. Clinical effect markers such as BP, levels of HbA_1c _and cholesterol were only assessed in three studies, showing small positive effects on BP in three, on HbA_1c _in two, and on cholesterol in one study. Since this systematic review, two additional RCTs of feedback in T2DM (one using benchmarking) were published [35,36]. However, neither of these studies showed any effect on either process or outcome measures. Therefore, while some studies suggest that feedback can lead to a small improvement in testing rates, overall it seems that it rarely changes downstream clinical outcomes such as HbA_1c _levels. The evidence from the literature therefore suggests that clinical inertia is not driven by physicians' knowledge deficit, but is due to reluctance among physicians to make management changes. Therefore, benchmarking, using comparisons with peer performance, could potentially add an emotional-competitive drive to overcome this barrier.

There are limited publications on the effects of benchmarking on quality of care in T2DM. A systematic review of controlled trials evaluating the effectiveness of various interventions to improve T2DM in primary care found that benchmarking improved diabetes care provision in settings characterised by poor overall standard of care at baseline [37]. However, clinical outcomes were rarely assessed, and no statistically significant improvements were demonstrated. A meta-analysis investigating benchmarking in clinical care found a small but significant positive effect on outcomes [38]. This analysis indicated that the effectiveness of benchmarking was improved when the benchmarked feedback was written rather than verbal or graphical, and when it was provided more frequently. In addition, providing combined group- and individual-level feedback appeared to have a positive effect.

There are few published reports of randomised studies investigating the effectiveness of benchmarking in T2DM in primary care. In a small study comparing two types of benchmarking interventions, 70 community physicians in the USA were randomly assigned to receive a multi-modal quality improvement intervention, including physician-specific benchmarking feedback or an identical intervention plus Achievable Benchmarks of Care (ABC) feedback [23]. In the physician-specific benchmarking feedback only group, the mean performance of the individual physicians on patient outcomes was compared with the performance of their anonymised peers. In the ABC feedback group, the individual performance of the physicians was compared with standards of excellence attained by top performers in the peer group. Odds ratios (ORs) for the ABC group vs comparison physicians who delivered appropriate care after the intervention were 1.57 (95% confidence interval [CI], 1.26,1.96) for influenza vaccination, 1.33 (95% CI, 1.05,1.69) for foot examination, and 1.33 (95% CI, 1.04,1.69) for long-term glucose control measurement. For serum cholesterol and TG, the effect of ABC feedback was not statistically significant. Another randomised trial that examined simultaneous benchmarking of US physicians and their patients failed to demonstrate improved safety or quality of care [36].

The paucity of controlled clinical trial evidence in T2DM provided the rationale for the initiation of OPTIMISE. The original protocol was developed in Belgium, based on a long-standing large scale feedback and benchmarking project in specialised diabetes centres, the 'Initiative for the Promotion of Quality and Epidemiology of Diabetes mellitus (IQED)' [39]. The OPTIMISE trial resulted from an interest to carry this initiative into the primary care setting. Because an intervention tested in different healthcare systems would provide greater information value, the OPTIMISE study was extended to five other countries in Europe. The main differences between the OPTIMISE design and the Belgian IQED initiative are that OPTIMISE was (i) performed in a primary care setting, (ii) utilised a RCT design with a comparative control group that did not receive benchmarking feedback, (iii) used a central laboratory, and (iv) focused on a smaller set of quality indicators. To minimise measurement variability, a central laboratory was identified for measuring HbA_1c _and lipids in the OPTIMISE trial. In real-life clinical practice, routine laboratory measurements could be used for benchmarking, provided the laboratories performing the analyses have suitable accredited quality assurance programmes, e.g. for HbA_1c _measurement [36]. Experience from the IQED initiative indicated that using a large dataset may somehow dilute the impact of benchmarking. The use of a limited number of critical quality indicators in OPTIMISE enabled the study investigators to provide benchmarking in a simple format, with succinct, easily interpretable messages accompanied by an immediately actionable item. In addition, the IQED initiative provided feedback every 18 months, while in OPTIMISE feedback was given every 4 months, as more frequent feedback was shown to augment the effect on outcomes [40].

The OPTIMISE trial has several strengths. Contrary to many studies in the field of quality improvement, this study uses a monofactorial intervention allowing its potential effects to be easily studied and contextualised. It randomises at the clinic level and not at the patient level to avoid confounding effects from physicians having both intervention and control patients. Results are analysed at the patient level as this is more clinically meaningful. Importantly, the RCT design avoids any positive effect bias that may be induced by increased attention caused by participation in a study, or by selecting more motivated physicians, or by overall changes in T2DM care over the study period. A possible confounder of the study may be the frequency of determining the quality indicators, which was pre-specified in the protocol. Frequency of testing may have increased for both groups of patients in the study compared with their testing frequency prior to study initiation. This alone may have led to an improvement in target attainment in both groups. One possible limitation of the study is that LDL-cholesterol levels were estimated using Friedewald's formula, rather than being directly measured. However, this reflects real-life clinical practice. A further limitation may be the requirement to establish a history of proteinuria from patient medical records, as proteinuria may not be routinely tested for and recorded. In addition to investigating the effects of benchmarking, the OPTIMISE study offers an opportunity to obtain comparable data on the quality of primary care received by patients with T2DM in six European countries. It is difficult to obtain comparable data across different European countries due to heterogeneity in data collection. Therefore, the OPTIMISE data have the potential to offer a particularly useful resource for European healthcare providers, and help inform diabetes care policy decisions at the European level along with other initiatives such as the European Core Indicators in Diabetes project (EUCID), which aims to create a platform to collect and analyse data on health status and care delivery for diabetes in Europe [41].

In summary, although feedback with benchmarking is a promising tool for quality improvement in chronic care in general, and in diabetes care specifically, there is a striking lack of clinical evidence from controlled trials. The results from OPTIMISE may have the potential to contribute to filling this gap.

## Competing interests

FN has received speaker fees, advisory board payments and has been provided with travel to scientific meetings by various pharmaceutical companies.

CB has received speaker fees and advisory board payments from various pharmaceutical companies.

ME has received speaker honoraria, consulting fees and research funding from AstraZeneca, Schering Plough, Pfizer, Solvay and Fournier, and has participated in clinical trials with AstraZeneca, Merck, Sanofi-Synthelabo, Solvay and Fournier,

EM has received speaker fees and advisory board payments from various pharmaceutical companies.

GM has received speaker fees, advisory board payments and has been provided with travel to scientific meetings by various pharmaceutical companies.

ND has received advisory board payments.

MH has served on an advisory panel and/or received speaker's honoraria or travel grants from Abbott, AstraZeneca, BMS, Boehringer Ingelheim, GSK, Eli Lilly, Menarini, Novartis, Novo Nordisk, sanofi aventis and Takeda.

## Authors' contributions

FN participated in the conception of the study, and helped to draft the manuscript. ND helped to draft the manuscript, and provided the literature analysis regarding the effect of feedback and benchmarking on the quality of diabetes care. CB participated in the discussion of results and drafting the manuscript. ME participated in the design of the study and in drafting the manuscript. EM participated in the conception of the study, and drafted the manuscript. GM participated in evaluating the study results and drafting the manuscript. MH was responsible for the conception of the study, and participated in its design and coordination and helped to draft the manuscript. All authors have read and approved the final manuscript.
